# The Looijenga–Lunts–Verbitsky Algebra and Verbitsky’s Theorem

**DOI:** 10.1007/s00032-022-00364-z

**Published:** 2022-10-03

**Authors:** Alessio Bottini

**Affiliations:** 1 Dipartimento di Matematica, Università di Roma Tor Vergata, Via della Ricerca Scientifica 1, 00133 Rome, Italy; 2 Université Paris-Saclay, CNRS, Laboratoire de Mathématiques d’Orsay, Rue Michel Magat, Bat. 307, 91405 Orsay, France

## Abstract

In these notes we review some basic facts about the LLV Lie algebra. It is a rational Lie algebra, introduced by Looijenga–Lunts and Verbitsky, acting on the rational cohomology of a compact Kähler manifold. We study its structure and describe one irreducible component of the rational cohomology in the case of a compact hyperkähler manifold.

## Introduction

### Motivation

In the papers [7] and [12] Looijenga–Lunts and Verbitsky introduce and study a Lie algebra ${\mathfrak {g}}_{\mathrm {tot}}(X)$ associated to a Kähler manifold *X*. It is defined as a Lie subalgebra of the graded endomorphism algebra ${{\,\mathrm{\mathrm {End}}\,}}(H^*(X,{\mathbb {Q}}))$ of the rational cohomology. Roughly speaking, if *X* is a hyperkähler manifold, the LLV algebra is generated by all the images of the $\mathfrak {sl}_2$-representations on $H^*(X,{\mathbb {Q}})$ coming from Kähler classes with respect to every complex structure on *X*.

Recently, the LLV algebra has found some interesting applications to the theory of hyperkähler manifolds. For example, in [4] the authors study how the cohomology of *X* decomposes in irreducible ${\mathfrak {g}}_{\mathrm {tot}}(X)$ representations. As a consequence, they compute Hodge numbers for hyperkähler manifolds of the known deformation type. See also [9] for more details on this.

Another striking application of the LLV algebra is due to Taelman [10]. He shows that an equivalence between the bounded derived categories of two hyperkähler varieties

$$\begin{aligned}\Phi : D^b(X) \xrightarrow {\sim } D^b(Y) \end{aligned}$$

induces an isomorphism between the corresponding LLV algebras. Moreover, the induced isomorphism $\Phi ^H: H^*(X,{\mathbb {Q}}) \xrightarrow {\sim } H^*(Y,{\mathbb {Q}})$ in cohomology is equivariant with respect to the natural actions. This implies the existence of a “rational Mukai lattice" preserved under derived equivalences. See [1] for an overview of these results.

### Basic Definitions

Let $V=\bigoplus _{k \in {\mathbb {Z}}} V_k$ be a finite dimensional graded vector space over a field ${\mathbb {F}}$ of characteristic 0, and denote by *h* the operator:

$$\begin{aligned} h|_{V_k}=k \cdot \mathrm {id}.\end{aligned}$$

#### Definition 1.1

Let  be a degree 2 endomorphism. We say *e* has the *Lefschetz property* ifis an isomorphism.

#### Remark 1.2

The degree two operators with the Lefschetz property form a Zariski open subset of ${{\,\mathrm{\mathrm {End}}\,}}_2(V)$.

#### Theorem 1.3

(Jacobson–Morozov, [6, Theorem 3]) An operator *e* has the Lefschetz property if and only if there exists a unique degree $-2$ endomorphism  such that

$$\begin{aligned}{}[e,f]=h.\end{aligned}$$

Moreover, if $L \subset {{\,\mathrm{\mathrm {End}}\,}}(V)$ is a semisimple Lie subalgebra and $e,h \in L$, then $f \in L$.

We say that the triple (*e*, *h*, *f*) is an $\mathfrak {sl}_2$-triple. The reason is that we can define a representation  of the Lie algebra $\mathfrak {sl}_2({\mathbb {F}})$ on the vector space *V* as followsIn the rest of these notes, we will mostly be interested in the graded rational vector space $V=H^{*}(X,{\mathbb {Q}})[N]$, where *X* is a compact Kähler manifold of dimension *N*. Here [*m*] indicates the shift by *m*, so that $V_0=H^N(X,{\mathbb {Q}})$. To any class $a \in H^2(X,{\mathbb {Q}})$ we can associate the operator in cohomology obtained by taking cup productThe operator *h* becomes

$$\begin{aligned} h|_{H^{k}(X,{\mathbb {Q}})} :=(k-N)\mathrm {id}.\end{aligned}$$

From Theorem 1.3 we see that if $e_{a}$ has the Lefschetz property (for example if *a* is a Kähler class), there is an operator $f_{a}$ of degree $-2$ that makes $(e_{a},h,f_{a})$ an $\mathfrak {sl}_2$-triple. Moreover, the map

$$\begin{aligned} f:H^2(X,{\mathbb {Q}}) \dashrightarrow {{\,\mathrm{\mathrm {End}}\,}}_{-2}(H^*(X,{\mathbb {Q}})), \end{aligned}$$

that sends *a* to the operator $f_{a}$ is defined on a Zariski open subset and rational.

#### Remark 1.4

If $a \in H^{1,1}(X,{\mathbb {Q}})$ is a Kähler class, it follows from standard Hodge theory that everything can be defined at the level of forms. The dual operator is $f_{a}=*^{-1}e_{a}*$, where $*$ is the Hodge star operator. The $\mathfrak {sl}_2$-action preserves the harmonic forms, so it induces an action on cohomology.

#### Definition 1.5

([7, 12]) Let *X* be a compact Kähler manifold. The *total Lie algebra* ${\mathfrak {g}}_{\mathrm {tot}}(X)$ of *X* is the Lie algebra generated by the $\mathfrak {sl}_2$-triples

$$\begin{aligned} (e_{a},h,f_{a}), \end{aligned}$$

where $a \in H^2(X,{\mathbb {Q}})$ is a class with the Lefschetz property.

The following is a general result about this Lie algebra for compact Kähler manifolds. Denote by $\phi$ the pairing on $H^*(X,{\mathbb {C}})$ given by

$$\begin{aligned} \phi (\alpha ,\beta ) = (-1)^q \int _X{\alpha .\beta }, \end{aligned}$$

if $\alpha$ has degree $N+2q$ or $N+2q+1$.

#### Proposition 1.6

([7, Proposition 1.6]) The Lie algebra ${\mathfrak {g}}_{\mathrm {tot}}(X)$ is semisimple and preserves $\phi$ infinitesimally. Moreover, the degree-0 part ${\mathfrak {g}}_{\mathrm {tot}}(X)_0$ is reductive.

**1.3** Now let *X* be a compact hyperkähler manifold of complex dimension 2*n*. In this case, the Lie algebra ${\mathfrak {g}}_{\mathrm {tot}}(X)$ is also called the Looijenga-Lunts-Verbitsky Lie algebra. It is well known that for each hyperkähler metric *g* on *X* we get an action of the quaternion algebra ${\mathbb {H}}$ on the real tangent bundle *TX*. This means that we have three complex structures *I*, *J*, *K* such that

1.1 $$\begin{aligned} IJ=-JI=K. \end{aligned}$$

To each of these complex structures we can associate Kähler forms $\omega _I :=g(I(-),-), \omega _J :=(J(-),-),\omega _K :=g(K(-),-)$ and holomorphic symplectic forms $\sigma _I=\omega _J + i \omega _K, \sigma _J=\omega _K +i \omega _I, \sigma _K=\omega _I+i\omega _J$.

#### Definition 1.7

The *characteristic* 3-*plane* *F*(*g*) of the metric *g* is

$$\begin{aligned} F(g) :=\langle [\omega _I],[\omega _J],[\omega _K] \rangle = \langle [\omega _I],[\Re \sigma _I],[\Im \sigma _I] \rangle \subset H^2(X,{\mathbb {R}}).\end{aligned}$$

#### Definition 1.8

([11]) Denote by ${\mathfrak {g}}_g \subset \mathrm {End}(H^*(X,{\mathbb {R}}))$ the Lie algebra generated by the $\mathfrak {sl}_2$-triples $(e_{a},h,f_{a})$ where $a \in F(g)$.

#### Remark 1.9

This Lie algebra is generated by the three $\mathfrak {sl}_2$-triples associated to the classes $[\omega _I],[\omega _J],[\omega _K]$. Indeed, from the discussion in the following section we will see that the subalgebra generated by these three $\mathfrak {sl}_2$-triples is semisimple. From the Jacobson-Morozov Theorem and the linearity of  we conclude that it contains every $\mathfrak {sl}_2$-triple $(e_{a},h,f_{a})$ with $a \in F(g)$.

## The Algebra Associated to a Metric

**2.1** In this section we study the smaller algebra ${\mathfrak {g}}_g$ and its action on cohomology. These results are due to Verbitsky [11], see also [5, Proposition 24.2], and [7, Proposition 4.4].

We start with a general algebraic construction. Let ${\mathbb {H}}$ be the quaternion algebra. As a real vector space it is generated by 1, *I*, *J*, *K*, where *I*, *J*, *K* satisfy the relations (1.1). We denote by ${\mathbb {H}}_0$ the pure quaternions, i.e. the linear combinations of *I*, *J*, *K*.

Let *V* be a left ${\mathbb {H}}$-module, equipped with an inner productand assume that *I*, *J*, *K* act on *V* via isometries. The ${\mathbb {H}}$-action gives three complex structures *I*, *J*, *K* on *V*, satisfying the relations (1.1). Consider the forms

$$\begin{aligned} \omega _I= & {} \langle I(-),- \rangle ,\\ \omega _J= & {} \langle J(-),- \rangle , \\ \omega _K= & {} \langle K(-),- \rangle \end{aligned}$$

and the holomorphic symplectic forms $\sigma _I=\omega _J + i \omega _K, \sigma _J=\omega _K +i \omega _I, \sigma _K=\omega _I+i\omega _J$.

### Remark 2.1

Note that the operators $e_{\lambda }$ for $\lambda =\omega _I,\omega _J,\omega _K$ have the Lefschetz property; the dual operator is given by $f_{\lambda }=*^{-1} e_{\lambda } *$, where $*$ is the Hodge star operator on $\Lambda ^{\bullet } V^*$ induced by the inner product.

### Definition 2.2

Let ${\mathfrak {g}}(V) \subset \mathrm {End}(\bigwedge ^{\bullet } V^*)$ be the Lie algebra generated by the $\mathfrak {sl}_2$-triples

$$\begin{aligned} (e_{\lambda },h,f_{\lambda })_{\lambda =\omega _I,\omega _J,\omega _K}, \end{aligned}$$

where *h* is the shifted degree operator.

In particular, this definition makes sense for the rank one module ${\mathbb {H}}$ equipped with the standard inner product. This gives a Lie algebra ${\mathfrak {g}}({\mathbb {H}}) \subset \mathrm {End}(\bigwedge ^{\bullet } {\mathbb {H}}^*).$ We denote by ${\mathfrak {g}}({\mathbb {H}})_0$ the degree-0 component of ${\mathfrak {g}}({\mathbb {H}})$ (here the degree is viewed as an endomorphism of the graded vector space). It is a Lie subalgebra, and we denote it by ${\mathfrak {g}}({\mathbb {H}})'_0 :=[{\mathfrak {g}}({\mathbb {H}})_0,{\mathfrak {g}}({\mathbb {H}})_0]$ its derived Lie algebra.

### Theorem 2.3

With the above notation we have the following.

1. There is a natural isomorphism ${\mathfrak {g}}(V) \simeq {\mathfrak {g}}({\mathbb {H}}).$
2. There is an isomorphism ${\mathfrak {g}}({\mathbb {H}}) \simeq \mathfrak {so}(4,1)$.
3. The algebra decomposes with respect to the degree as
  $$\begin{aligned} {\mathfrak {g}}({\mathbb {H}})={\mathfrak {g}}({\mathbb {H}})_{-2} \oplus {\mathfrak {g}}({\mathbb {H}})_0 \oplus {\mathfrak {g}}({\mathbb {H}})_{2}.\end{aligned}$$
  Furthermore, ${\mathfrak {g}}({\mathbb {H}})_{\pm 2} \simeq {\mathbb {H}}_0$ as Lie algebras, and ${\mathfrak {g}}({\mathbb {H}})_0={\mathfrak {g}}({\mathbb {H}})'_0 \oplus {\mathbb {R}}h$ with ${\mathfrak {g}}({\mathbb {H}})'_0 \simeq {\mathbb {H}}_0$; this last isomorphism is compatible with the actions on $\bigwedge ^{\bullet } V^*$.

### Proof

(1) Since $\langle -,- \rangle$ is ${\mathbb {H}}$-invariant, we can find an orthogonal decomposition

$$\begin{aligned} V = {\mathbb {H}} \oplus \dots \oplus {\mathbb {H}}. \end{aligned}$$

Taking exterior powers we get $\bigwedge ^{\bullet } V^* = \bigwedge ^{\bullet } {\mathbb {H}}^* \otimes \dots \otimes \bigwedge ^{\bullet } {\mathbb {H}}^*$. This gives an injective map , given by the natural tensor product representation. It is a direct check that the image of this morphism is exactly the algebra ${\mathfrak {g}}(V)$.

(2) Consider the subrepresentation $W \subset \bigwedge ^{\bullet } {\mathbb {H}}^*$ given by

$$\begin{aligned} W = \bigwedge ^{0} {\mathbb {H}}^* \oplus \langle \omega _I,\omega _J,\omega _K \rangle \oplus \bigwedge ^{4}{\mathbb {H}}^*. \end{aligned}$$

We equip it with the quadratic form given by setting $\bigwedge ^{0} {\mathbb {H}}^* \oplus \bigwedge ^{4}{\mathbb {H}}^*$ to be a hyperbolic plane, orthogonal to the 3-plane, and $\{\omega _I,\omega _J,\omega _K\}$ to be an orthonormal basis of the 3-plane. By a direct computation we can see that the action of ${\mathfrak {g}}({\mathbb {H}})$ respects infinitesimally this quadratic form. This gives a map2.1that we next show to be an isomorphism.

Since *W* has dimension 5, the Lie algebra $\mathfrak {so}(W)$ has dimension 10. Now consider the following 10 elements of ${\mathfrak {g}}({\mathbb {H}})$:

$$\begin{aligned} h,e_I,e_J,e_K,f_I,f_J,f_K,K_{IJ},K_{IK},K_{JK}, \end{aligned}$$

where $K_{IJ} :=[e_I,f_J], K_{IK}=[e_I,f_K]$ and $K_{JK}=[e_J,f_K]$. Verbitsky [11] showed that $K_{IJ}$ acts like the Weil operator associated with the Hodge structure on $\bigwedge ^{\bullet }{\mathbb {H}}^*$ given by *K*, and similarly $K_{JK}$ and $K_{IK}$. This means that it acts on a (*p*, *q*) form with respect to *K* as multiplication by $i(p-q)$. It follows that the ten operators above are linearly independent over *W*, hence the map is surjective. Moreover they generate ${\mathfrak {g}}({\mathbb {H}})$ as a vector space. In fact, they generate ${\mathfrak {g}}({\mathbb {H}})$ as a Lie algebra, and one has the following relations (see [11]):

$$\begin{aligned} {}[K_{\lambda ,\mu },K_{\mu ,\nu }]=K_{\lambda ,\nu },&\quad [K_{\lambda ,\mu },h] = 0, \\ {}[K_{\lambda ,\mu },e_{\mu }]=2e_{\lambda },&\quad [K_{\lambda ,\mu },f_{\mu }]=2f_{\lambda }, \\ {}[K_{\lambda ,\mu },e_{\nu }]=0,&\quad [K_{\lambda ,\mu },f_{\nu }]=0, \end{aligned}$$

where $\lambda ,\mu ,\nu \in \{I,J,K\}$ and $\nu \ne \lambda , \nu \ne \mu$. This implies that they are a basis of ${\mathfrak {g}}({\mathbb {H}})$, hence the map (2.1) is an isomorphism.

Point (3) follows using this explicit basis. Indeed we have

$$\begin{aligned} {\mathfrak {g}}({\mathbb {H}})_{-2}&= \langle f_I,f_J,f_K \rangle , \quad {\mathfrak {g}}({\mathbb {H}})_{2} = \langle e_I,e_J,e_K \rangle , \ \text {and} \\ {\mathfrak {g}}({\mathbb {H}})_{0}&= \langle K_{IJ},K_{JK},K_{IK} \rangle \oplus {\mathbb {R}}h. \\ \end{aligned}$$

In particular, we have:Since $I,J,K \in {\mathbb {H}}_0$ act on $\bigwedge ^{\bullet }{\mathbb {H}}^*$ as Weil operators for the corresponding complex structures on ${\mathbb {H}}$, the isomorphism is compatible with the actions. $\square$

Now we can compute the Lie algebra ${\mathfrak {g}}_g$. As above we denote by $({\mathfrak {g}}_g)_0$ the degree-0 part, and by $({\mathfrak {g}}_g)'_0 :=[({\mathfrak {g}}_g)_0,({\mathfrak {g}}_g)_0]$ its derived Lie algebra.

### Proposition 2.4

Let (*X*, *g*) be a hyperkähler manifold with a fixed hyperkähler metric.

1. There is a natural isomorphism of graded Lie algebras ${\mathfrak {g}}_g \simeq {\mathfrak {g}}({\mathbb {H}})$. In particular ${\mathfrak {g}}_g \simeq \mathfrak {so}(4,1)$.
2. The semisimple part $({\mathfrak {g}}_g)'_0$ acts on $H^*(X,{\mathbb {R}})$ via derivations.

### Proof

(1). Consider the Lie subalgebra $\hat{{\mathfrak {g}}}_{g} \subset {{\,\mathrm{\mathrm {End}}\,}}(\Omega ^{\bullet }_X)$, generated by the $\mathfrak {sl_2}$-triples $(e_a,h,f_a)$ with $a \in F(g)$, at the level of forms (in particular $f_a=*^{-1}e_a*$). From Theorem 2.3, we see that for every point $x \in X$ there is an inclusion . This gives an inclusion . It follows from the definitions that the two algebras ${\mathfrak {g}}({\mathbb {H}})$ and $\hat{{\mathfrak {g}}}_{g}$ are equal as subalgebras of $\prod _{x \in X} {{\,\mathrm{\mathrm {End}}\,}}(\Omega ^{\bullet }_{X,x})$.

Since the metric *g* is fixed, the $\mathfrak {sl}_2$-triples $(e_a,h,f_a)$ preserve the harmonic forms ${\mathcal {H}}^*(X)$, and so does $\hat{{\mathfrak {g}}}_{g}$. Since ${\mathcal {H}}^*(X) \simeq H^*(X,{\mathbb {R}})$ we get a morphismThis map is surjective, because the image contains the $\mathfrak {sl}_2$-triples that generate ${\mathfrak {g}}_g$. Moreover, by explicit computations similar to the proof of Theorem 2.3, we can see that $\dim {\mathfrak {g}}_g \ge 10$. Hence the map is an isomorphism.

(2). From the previous proposition we have an isomorphism compatible with the actions on cohomology

$$\begin{aligned}({\mathfrak {g}}_g)'_0 \simeq {\mathfrak {g}}({\mathbb {H}})'_0 \simeq {\mathbb {H}}_0.\end{aligned}$$

Hence, it suffices to prove the statement for the action of *I*, *J*, *K*. Each of them gives a complex structure, and acts as the Weil operator on the associated Hodge decomposition. So, the action on (*p*, *q*) forms is given by multiplication by $i(p-q)$, which is a derivation. $\square$

## The Total Lie Algebra

The goal of this section is to prove the following result due to Looijenga and Lunts [7, Proposition 4.5] and Verbitsky [12, Theorem 1.6].

### Theorem 3.1

Let *X* be a hyperkähler manifold. With the above notation, we have the following.

1. The total Lie algebra ${\mathfrak {g}}_{\mathrm {tot}}(X)$ lives only in degrees $-2,0,2$, so it decomposes as:
  $$\begin{aligned} {\mathfrak {g}}_{\mathrm {tot}}(X)={\mathfrak {g}}_{\mathrm {tot}}(X)_{-2} \oplus {\mathfrak {g}}_{\mathrm {tot}}(X)_0 \oplus {\mathfrak {g}}_{\mathrm {tot}}(X)_2. \end{aligned}$$
2. There are canonical isomorphisms ${\mathfrak {g}}_{\mathrm {tot}}(X)_{\pm 2} \simeq H^2(X,{\mathbb {Q}})$.
3. There is a decomposition ${\mathfrak {g}}_{\mathrm {tot}}(X)_0 = {\mathfrak {g}}_{\mathrm {tot}}(X)_0' \oplus {\mathbb {Q}}h$ with ${\mathfrak {g}}_{\mathrm {tot}}(X)_0' \simeq \mathfrak {so}(H^2(X,{\mathbb {Q}}),q)$, where *q* is the Beauville–Bogolomov–Fujiki quadratic form, see [2]. Furthermore ${\mathfrak {g}}_{\mathrm {tot}}(X)_0'$ acts on $H^*(X,{\mathbb {Q}})$ by derivations.

The main geometric input in the proof is the following lemma.

### Lemma 3.2

If *X* is a compact hyperkähler manifold, then $[f_a,f_b]=0$ for every $a,b \in H^2(X,{\mathbb {R}})$ for which *f* is defined.

The proof relies on the following fact.

### Proposition 3.3

The set of characteristic 3-planes is open in the Grassmannian of 3-planes in $H^2(X,{\mathbb {R}})$.

In turn, this follows from a celebrated theorem by Yau.

### Theorem 3.4

(Yau) Let *X* be a hyperkähler manifold, and let *I* be a complex structure on *X*. If $\omega$ is a Kähler class, then there is a unique hyperkähler metric *g* such that $[\omega _I]=\omega .$

### Proof of Lemma 3.2

If we fix a hyperkähler metric *g* on *X*, then for every $a,b \in F(g)$ we have $[f_a,f_b]=0$. This holds already at the level of forms, using the definition $f_a=*^{-1}e_a*$ and the fact that $*$ depends only on the metric. Let $a \in H^2(X,{\mathbb {R}})$ be a class for which $f_a$ is defined. Since *f* is a rational endomorphism, the condition $[f_a,f_b]=0$ is Zariski closed with respect to $b \in H^2(X,{\mathbb {R}})$. From Proposition 3.3 it follows that the set

$$\begin{aligned} \{ b \in H^2(X,{\mathbb {R}}) \mid a,b \in F(g) \text { for some metric } g \} \end{aligned}$$

is open. Since $[f_a,f_b]=0$ for every *b* in this open set, we get $[f_a,f_b]=0$ for every *b* where $f_b$ is defined. $\square$

While the statement of Theorem 3.1 is over ${\mathbb {Q}}$, we will give the proof over ${\mathbb {R}}$ following [7].

### Proof of Theorem 3.1

Consider the subspace

$$\begin{aligned}V :=V_{-2} \oplus V_0 \oplus V_2 \subset {\mathfrak {g}}_{\mathrm {tot}}(X),\end{aligned}$$

where $V_2$ is the abelian Lie subalgebra generated by $e_{a}$ with $a \in H^2(X,{\mathbb {R}})$, $V_{-2}$ is the abelian Lie subalgebra generated by the $f_{a}$ with $a \in H^2(X,{\mathbb {R}})$ where $f_a$ is defined, and $V_0$ is the Lie subalgebra generated by $[e_{a},f_{b}]$. To prove (1) and (2), it is enough to show that *V* is a Lie subalgebra of ${\mathfrak {g}}_{\mathrm {tot}}(X)$. Indeed, since ${\mathfrak {g}}_{\mathrm {tot}}(X)$ is generated by elements contained in *V* this would imply $V= {\mathfrak {g}}_{\mathrm {tot}}(X)$. Since $V_2$ and $V_{-2}$ are abelian, it suffices to show that $[V_0,V_2] \subset V_2$ and $[V_0,V_{-2}] \subset V_{-2}$.

### Claim

Define $V'_0 :=[V_0,V_0]$. We have $V_0=V_0' \oplus {\mathbb {R}}h$ where $V_0'$ acts on cohomology via derivations.

### Proof of the claim

Proposition 3.3 implies that the set $\{(a,b) \in H^2(X,{\mathbb {R}}) \times H^2(X,{\mathbb {R}}) \mid a,b \!\in \! F(g) \text { for some metric } g \}$ is open. Arguing as in the proof of Lemma 3.2 we see that $V_0$ is generated by the elements $[e_a,f_b]$ with $a,b \in F(g)$ for some metric *g*. If we fix a hyperkähler metric *g*, the elements $[e_a,f_b]$ with $a,b \in F(g)$ generate the Lie algebra $({\mathfrak {g}}_g)_0$ and their brackets the Lie subalgebra $({\mathfrak {g}}_g)'_0$. Thus, $V'_0$ is generated by the Lie algebras $({\mathfrak {g}}_g)'_0$ and their brackets. Since the Lie algebras $({\mathfrak {g}}_g)'_0$ act on cohomology via derivations, the same is true for their brackets, hence $V'_0$ acts via derivations. Moreover, from point (3) of Theorem 2.3 we get the decomposition $V_0=V_0' + {\mathbb {R}}h$. Since ${\mathfrak {g}}_{\mathrm {tot}}(X)_0$ is reductive (Proposition 1.6) and *h* is in the center, we get $h \not \in V_0' \subset {\mathfrak {g}}_{\mathrm {tot}}(X)'_0$, so the sum is direct. $\square$

Now we show that $[V_0,V_2] \subset V_2$. Since the adjoint action of *h* gives the grading, it is enough to show that $[V_0',V_2] \subset V_2$. Let $u \in V_0'$ and $e_a \in V_2$. For every $x \in H^2(X,{\mathbb {R}})$ we have

3.1 $$\begin{aligned}{}[u,e_a](x) = u(a.x) -a.u(x) = u(a).x=e_a(x), \end{aligned}$$

because *u* is a derivation.

The inclusion $$[V_0,V_{-2}] \subset V_{-2}$$ is more difficult. Let $$G'_0 \subset GL(H^*(X,{\mathbb {R}}))$$ be the closed Lie subgroup with Lie algebra $$V'_0$$. For every $$t \in G'_0$$ we have $$t e_a t^{-1} = e_{t(a)}$$ and $$t h t^{-1} =h $$, by integrating the analogous relations at the level of Lie algebras. Since the third element of a $$\mathfrak {sl_2}$$-triple is unique, we get that $$t f_a t^{-1} = f_{t(a)}$$. This implies that the adjoint action of $$G'_0$$ leaves $$V_{-2}$$ invariant, hence so does the Lie algebra $$V'_0$$.

To summarize, at this point we showed (1) and (2), and also that ${\mathfrak {g}}_{\mathrm {tot}}(X)'_0$ acts via derivations. It remains to show that ${\mathfrak {g}}_{\mathrm {tot}}(X)'_0 \simeq \mathfrak {so}(H^2(X,{\mathbb {R}}),q)$.

We begin by defining the map . For this, we consider the restriction of the action of ${\mathfrak {g}}_{\mathrm {tot}}(X)'_0$ to $H^2(X,{\mathbb {R}})$, and show that it preserves infinitesimally the Beauville–Bogomolov–Fujiki form *q*. We can fix a hyperkähler metric *g* and check this for $({\mathfrak {g}}_g)'_0$, because these Lie subalgebras generate ${\mathfrak {g}}_{\mathrm {tot}}(X)'_0$. From Theorem 2.3 it is enough to check it for the Weil operators associated to the three complex structures *I*, *J*, *K* induced from *g*. Fix one of them, say *I*; we have to verify that

$$\begin{aligned} q(I\alpha ,\beta ) + q(\alpha , I \beta ) = 0,\end{aligned}$$

for every $\alpha ,\beta \in H^2(X,{\mathbb {R}})$. This follows from a direct verification using the *q*-orthogonal decomposition

$$\begin{aligned} H^2(X,{\mathbb {R}}) = (H^{2,0}(X) \oplus H^{0,2}(X)) \cap H^2(X,{\mathbb {R}}) \oplus H^{1,1}(X,{\mathbb {R}}), \end{aligned}$$

induced by the Hodge decomposition with respect to the complex structure *I*.

To conclude the proof it remains to show that this map is bijective; we begin with the surjectivity. Fix a hyperkähler metric *g*, the image of the Lie algebra $({\mathfrak {g}}_g)'_0$ in $\mathfrak {so}(H^2(X,{\mathbb {R}}),q)$ is generated (as a vector space) by the Weil operators associated to *I*, *J*, *K*. Using this, it is easy to see that $({\mathfrak {g}}_g)'_0$ kills the *q*-orthogonal complement to the characteristic 3-plane *F*(*g*), and it maps onto $\mathfrak {so}(F(g),q|_{F(g)})$. One can check that varying the metric *g* the Lie subalgebras $\mathfrak {so}(F(g),q|_{F(g)})$ generate $\mathfrak {so}(H^2(X,{\mathbb {R}}))$, hence the surjectivity.

For the injectivity we proceed as follows. Let $SH^2(X,{\mathbb {R}}) \subset H^*(X,{\mathbb {R}})$ be the graded subalgebra generated by $H^2(X,{\mathbb {R}})$; it is a ${\mathfrak {g}}_{\mathrm {tot}}(X)$ representation for Corollary 4.6. By Lemma 4.7, the map  is injective. Since ${\mathfrak {g}}_{\mathrm {tot}}(X)'_0$ acts via derivations, the map must be injective already at the level of $H^2(X,{\mathbb {R}})$. $\square$

### Definition 3.5

We define the *Mukai completion* of the quadratic vector space $(H^2(X,{\mathbb {Q}}),q)$ as the quadratic vector space

$$\begin{aligned} (\tilde{H}(X,{\mathbb {Q}}),\tilde{q}) :=(H^2(X,{\mathbb {Q}}),q) \oplus U\end{aligned}$$

where *U* is the quadratic vector space ${\mathbb {Q}}^2$ with the quadratic form $$\begin{pmatrix} 0 &{}\quad 1 \\ 1 &{}\quad 0 \end{pmatrix}$$

### Corollary 3.6

There is a canonical isomorphism

$$\begin{aligned} {\mathfrak {g}}_{\mathrm {tot}}(X) \simeq \mathfrak {so}(\tilde{H}(X,{\mathbb {Q}}),\tilde{q}). \end{aligned}$$

### Proof

Recall that for a rational quadratic space (*V*, *q*) there is an isomorphismThe desired isomorphism follows from this, at least at the level of vector spaces. The computations to show that it is in fact an isomorphism of Lie algebras are carried out in [4, Proposition 2.7]. $\square$

### Example 3.7

If *X* is a *K*3 surface, then the Mukai completion ${\tilde{H}}(X,{\mathbb {Q}})$ is the rational cohomology $H^*(X,{\mathbb {Q}})$ with the usual Mukai pairing. This identification is compatible with the action of ${\mathfrak {g}}_{\mathrm {tot}}(X)$.

### Corollary 3.8

The Hodge structure on $H^*(X,{\mathbb {R}})$ is determined by the Hodge structure on $H^2(X,{\mathbb {R}})$ and by the action of ${\mathfrak {g}}_{\mathrm {tot}}(X)_{2,{\mathbb {R}}} \simeq H^2(X,{\mathbb {R}})$ on $H^*(X,{\mathbb {R}})$.

### Proof

Let *I*, *J*, *K* be the three natural complex structures associated to a hyperkähler metric *g*, and assume *I* is the given one. As recalled before, the commutator $K_{JK}=[e_{J},f_{K}]$ acts like the Weil operator for *I*; hence it recovers the Hodge structure. By definition, it depends only on the classes $[\omega _I],[\omega _K]$ and their action on $H^*(X,{\mathbb {R}})$. Since the Hodge structure is given by the class of the symplectic form $[\sigma _I]=[\omega _J]+i[\omega _K]$, the conclusion follows. $\square$

Recall that if ${\mathfrak {g}}$ is a Lie algebra, the *universal enveloping algebra* $U{\mathfrak {g}}$ of ${\mathfrak {g}}$ is the smallest associative algebra extending the bracket on ${\mathfrak {g}}$. It is defined as the quotient of the tensor algebra by the elements of the form:

$$\begin{aligned} x \otimes y -y \otimes x -[x,y] \quad x,y \in {\mathfrak {g}}.\end{aligned}$$

In particular, if ${\mathfrak {g}}$ is abelian, then $U{\mathfrak {g}}=\mathrm {Sym}^*{\mathfrak {g}}$.

### Corollary 3.9

There is a natural decomposition

$$\begin{aligned} U{\mathfrak {g}}_{\mathrm {tot}}(X)=U{\mathfrak {g}}_{\mathrm {tot}}(X)_{2} \cdot U{\mathfrak {g}}_{\mathrm {tot}}(X)_0 \cdot U{\mathfrak {g}}_{\mathrm {tot}}(X)_{-2},\end{aligned}$$

where $\cdot$ denotes the multiplication in $U{\mathfrak {g}}_{\mathrm {tot}}(X)$.

### Proof

We have to show that every element in $x \in U{\mathfrak {g}}_{\mathrm {tot}}(X)$ can be written as a sum of elements of the form $x_{2} \cdot x_0 \cdot x_{-2}$ with $x_i \in U{\mathfrak {g}}_{\mathrm {tot}}(X)_i$. It is enough to check this on the images of pure tensors. On these it follows from the fact that the bracket is graded and the decomposition in Theorem 3.1. $\square$

## Primitive Decomposition

In this section, we study the relationship between the actions of ${\mathfrak {g}}_{\mathrm {tot}}(X)$ and ${\mathfrak {g}}_{\mathrm {tot}}(X)_0$ on $H^*(X,{\mathbb {Q}})$, where *X* is a compact hyperkähler manifold of dimension $\text {dim}(X)=2n$. The main reference is [7], see also [8, Theorem 4.4].

### Definition 4.1

Let *V* be a ${\mathfrak {g}}_{\mathrm {tot}}(X)$-representation. We define the primitive subspace as:

$$\begin{aligned} \mathrm {Prim}(V)=\{ x \in V \mid ({\mathfrak {g}}_{\mathrm {tot}}(X)_{-2}).x=0 \}.\end{aligned}$$

If $V=H^*(X,{\mathbb {Q}})$ is the standard representation we denote the primitive subspace as $\mathrm {Prim}(X)$.

### Remark 4.2

This definition is compatible with the usual notion of primitive element with respect to a Kähler class $\alpha$ in Hodge theory. Indeed, by Lemma 6.24 in [14] we see that an element $x \in H^k(X,{\mathbb {R}})$ is primitive with respect to $\alpha$ if and only if it is killed by the dual operator $f_\alpha$.

### Remark 4.3

The primitive subspace $\mathrm {Prim}(V)$ is a ${\mathfrak {g}}_{\mathrm {tot}}(X)_0$-subrepresentation. This follows from the fact that $[{\mathfrak {g}}_{\mathrm {tot}}(X)_0,{\mathfrak {g}}_{\mathrm {tot}}(X)_{-2}] \subset {\mathfrak {g}}_{\mathrm {tot}}(X)_{-2}$.

### Definition 4.4

The *Verbitsky component* $SH^2(X,{\mathbb {Q}}) \subseteq H^*(X,{\mathbb {Q}})$ is the graded subalgebra generated by $H^2(X,{\mathbb {Q}})$.

### Proposition 4.5

([7, Corollary 1.13 and Corollary 2.3]) The cohomology $H^*(X,{\mathbb {Q}})$ is generated by $\mathrm {Prim}(X)$ as a $SH^2(X,{\mathbb {Q}})$-module. Moreover, if $W \subset \mathrm {Prim}(X)$ is a ${\mathfrak {g}}_{\mathrm {tot}}(X)_0$ irreducible subrepresentation, then $SH^2(X,{\mathbb {Q}}).W \subset H^*(X,{\mathbb {Q}})$ is an irreducible ${\mathfrak {g}}_{\mathrm {tot}}(X)$-module.

### Proof

Since ${\mathfrak {g}}_{\mathrm {tot}}(X)$ is semisimple, we can decompose the cohomology in irreducible ${\mathfrak {g}}_{\mathrm {tot}}(X)$-representations:

$$\begin{aligned} H^*(X,{\mathbb {Q}}) = V_1 \oplus \dots \oplus V_k.\end{aligned}$$

The primitive part is compatible with this decomposition, so we get the decomposition

$$\begin{aligned} \mathrm {Prim}(X) = \mathrm {Prim}(V_1) \oplus \dots \oplus \mathrm {Prim}(V_k),\end{aligned}$$

of $${\mathfrak {g}}_{\mathrm {tot}}(X)_0$$-representations.

We first want to show that $SH^2(X,{\mathbb {Q}}).\mathrm {Prim}(V_i)=V_i$. We have

4.1 $$\begin{aligned} SH^2(X,{\mathbb {Q}}).\mathrm {Prim}(V_i)=U{\mathfrak {g}}_{\mathrm {tot}}(X)_{2}.\mathrm {Prim}(V_i) =U{\mathfrak {g}}_{\mathrm {tot}}(X).\mathrm {Prim}(V_i) \subset V_i, \end{aligned}$$

where the first equality follows from the fact that ${\mathfrak {g}}_{\mathrm {tot}}(X)_{2}$ is abelian, and the second from Corollary 3.9 and Remark 4.3. Thus $SH^2(X,{\mathbb {Q}}).\mathrm {Prim}(V_i)$ is a ${\mathfrak {g}}_{\mathrm {tot}}(X)$ subrepresentation of $V_i$, but $V_i$ is irreducible, so the equality holds. This proves the first part of the proposition.

To prove the second part it is enough to show that each $\mathrm {Prim}(V_i)$ is irreducible as a ${\mathfrak {g}}_{\mathrm {tot}}(X)_0$-representation. Assume it is not and write $\mathrm {Prim}(V_i)=W_1 \oplus W_2$. The identities (4.1) show that acting with $SH^2(X,{\mathbb {Q}})$ gives a decomposition $V_i=SH^2(X,{\mathbb {Q}}).W_1 \oplus SH^2(X,{\mathbb {Q}}).W_2$. Again, this contradicts the fact that $V_i$ is an irreducible ${\mathfrak {g}}_{\mathrm {tot}}(X)$-representation. $\square$

### Corollary 4.6

The Verbitsky component $SH^2(X,{\mathbb {Q}}) \subset H^*(X,{\mathbb {Q}})$ is an irreducible ${\mathfrak {g}}_{\mathrm {tot}}(X)$ subrepresentation.

### Proof

By definition we have $SH^2(X,{\mathbb {Q}})=SH^2(X,{\mathbb {Q}}).H^0(X,{\mathbb {Q}})$, and $H^0(X,{\mathbb {Q}}) \subset \mathrm {Prim}(X)$. So it is enough to observe that $H^0(X,{\mathbb {Q}})$ is preserved by ${\mathfrak {g}}_{\mathrm {tot}}(X)_0$, then we conclude by the previous proposition. $\square$

### Lemma 4.7

The restriction map  is injective.

### Proof

Let $K \subset {\mathfrak {g}}_{\mathrm {tot}}(X)_{{\mathbb {R}}}$ be the kernel. It is immediate to see that $K \subset {\mathfrak {g}}_{\mathrm {tot}}(X)'_0$. The action of *K* is 0 on $H^2(X,{\mathbb {R}})$, so by (3.1) we get $[K,{\mathfrak {g}}_{\mathrm {tot}}(X)_{{\mathbb {R}},2}]=0$. Taking the Lie group of *K* and the corresponding adjoint action, we see that $[K,f_a]=0$ for every $a \in H^2(X,{\mathbb {R}})$ for which $f_a$ is defined. So *K* has bracket 0 with ${\mathfrak {g}}_{\mathrm {tot}}(X)_{{\mathbb {R}},2}$ and ${\mathfrak {g}}_{\mathrm {tot}}(X)_{{\mathbb {R}},-2}$, thus also with ${\mathfrak {g}}_{\mathrm {tot}}(X)_{{\mathbb {R}},0}$. Since ${\mathfrak {g}}_{\mathrm {tot}}(X)$ is semisimple this implies *K*=0. $\square$

## Verbitsky’s Theorem

In this section we give a proof of a result by Verbitsky on the structure of the irreducible component $SH^2(X)$. This result is particularly useful to study the action of the LLV algebra on the rational cohomology. As a consequence, one can understand $SH^2(X)$ as a highest weight module for ${\mathfrak {g}}_{\mathrm {tot}}(X)$, see [9]. The argument presented here was given by Bogomolov in [3].

### Theorem 5.1

There is a natural isomorphism of algebras and ${\mathfrak {g}}_{\mathrm {tot}}(X)_0$-representations:

$$\begin{aligned} SH^2(X,\mathbb {{\mathbb {C}}}) \simeq \mathrm {Sym}^*H^2(X,{\mathbb {C}})/\langle \alpha ^{n+1} \mid q(\alpha )=0 \rangle .\end{aligned}$$

The key technical fact is the following lemma from representation theory, of which we omit the proof.

### Lemma 5.2

Denote by *A* the graded ${\mathbb {C}}$-algebra $\mathrm {Sym}^*H^2(X,{\mathbb {C}})/\langle \alpha ^{n+1} \mid q(\alpha )=0 \rangle$. Then we have:

1. $$A_{2n} \simeq {\mathbb {C}}$$.
2. The multiplication map  induces a perfect pairing.

### Proof of the theorem

From the local Torelli Theorem we have that $\alpha ^{n+1}=0$ for an open subset of the quadric $\{ \alpha \in H^2(X,{\mathbb {C}}) \mid q(\alpha )=0 \}$. Since the condition $\alpha ^{n+1}=0$ is Zariski closed, we get that it holds for the entire quadric. Consider the multiplication mapThe kernel contains $\{ \alpha ^{n+1} \mid q(\alpha )=0 \}$, hence it factors via the ring *A*. It is an algebra homomorphism by construction, and a map of ${\mathfrak {g}}_{\mathrm {tot}}(X)_0$-representations because ${\mathfrak {g}}_{\mathrm {tot}}(X)'_0$ acts via derivations.

The induced map  is surjective by construction. If it were not injective, by the above lemma, the kernel would contain $A_{2n}$. But this is impossible, because in top degree the map  is non-zero. Indeed if $\sigma$ is a holomorphic symplectic form, the form $(\sigma +{\overline{\sigma }})^{2n}$ is non-zero. $\square$

### Corollary 5.3

There are natural isomorphisms defined over ${\mathbb {Q}}$

$$\begin{aligned} SH^2(X,{\mathbb {Q}})_{2k} \simeq \left\{ \begin{array}{ll} \mathrm {Sym}^{k} H^2(X,{\mathbb {Q}}) &{}\quad \text {if } k\le n, \\ \mathrm {Sym}^{2n-k} H^2(X,{\mathbb {Q}})&{}\quad \text {if } n < k \le 2n. \end{array}\right. \end{aligned}$$

### Proof

From Theorem 5.1 it follows that the properties (1) and (2) in Lemma 5.2 hold for $SH^2(X,{\mathbb {C}})$. Up to up to multiplication with a nonzero scalar, they also hold for $SH^2(X,{\mathbb {Q}})$. The multiplication map  is an isomorphism if $k \le n$, because it is so over ${\mathbb {C}}$. If $k>n$ we have

$$\begin{aligned} SH^2(X,{\mathbb {Q}})_{2k} \simeq SH^2(X,{\mathbb {Q}})^*_{4n-2k} \simeq \mathrm {Sym}^{2n-k} H^2(X,{\mathbb {Q}})^* \simeq \mathrm {Sym}^{2n-k} H^2(X,{\mathbb {Q}}),\end{aligned}$$

where the last equality is due to the Beauville–Bogomolov–Fujiki form. $\square$

### Example 5.4

If *X* is of $\mathrm {K3}^{[2]}$-type, for dimensional reasons, the Verbitsky component *SH*(*X*) is the only irreducible component in the cohomology. For higher values of *n* the decomposition of $H^*(X,{\mathbb {Q}})$ in irreducible components is described in [4], for more details on this see [9].

## Spin Action

In this section we study how the action of $\mathfrak {so}(H^2(X,{\mathbb {Q}}),q)$ integrates to an action of the simply connected algebraic group $\underline{\mathrm {Spin}}(H^2(X,{\mathbb {Q}}),q)$. Recall that there is an exact sequence of algebraic groupsFor more information see [1] and [9].

### Proposition 6.1

([8, Theorem 4.4], [13]) The action of $\mathfrak {so}(H^2(X,{\mathbb {Q}}),q)$ on $H^*(X,{\mathbb {Q}})$ integrates to an action of the algebraic group $\underline{\mathrm {Spin}}(H^2(X,{\mathbb {Q}}),q)$ via ring isomorphisms. On the even cohomology it induces an action of $\underline{\mathrm {SO}}(H^2(X,{\mathbb {Q}}),q)$.

### Proof

The first part of the statement is clear: we can always lift the action because the algebraic group $\underline{\mathrm {Spin}}(H^2(X,{\mathbb {Q}}),q)$ is simply connected. The group $\underline{\mathrm {Spin}}(H^2(X,{\mathbb {Q}}),q)$ acts via ring isomorphisms because the Lie algebra acts via derivations.

To show the second part of the statement we proceed as follows. Fix a hyperkähler metric *g* and a compatible complex structure *I*. The Weil operator with respect to *I* is contained in $({\mathfrak {g}}_g)'_0 \simeq \mathfrak {so}(H^2(X,{\mathbb {Q}}))$. The exponential $\exp (\pi I) \in \underline{\mathrm {Spin}}(H^2(X,{\mathbb {Q}}),q)$ acts on the (*p*, *q*) part of $H^k(X,{\mathbb {C}})$ as multiplication by $e^{i(p-q)\pi }$, which is just multiplication by $(-1)^k$. In particular, on $H^2(X,{\mathbb {Q}})$ it acts as the identity, so $\exp (\pi I)=-1 \in \underline{\mathrm {Spin}}(H^2(X,{\mathbb {Q}}),q)$. We have also shown that $-1 \in \underline{\mathrm {Spin}}(H^2(X,{\mathbb {Q}}),q)$ acts on $H^k(X,{\mathbb {Q}})$ as $(-1)^k$, which means that the action on even cohomology factors through $\underline{\mathrm {SO}}(H^2(X,{\mathbb {Q}}),q)$. $\square$

## References

[1] Beckmann, T.: Derived categories of hyper-Kähler varieties via the LLV algebra (2021) 10.1007/s00032-022-00358-x PMC9708817 36466317

[2] Beri, P., Debarre, O.: On the Hodge and Betti numbers of hyper-Kähler manifolds (2021)

[3] Bogomolov F (1996). On the cohomology ring of a simple hyper-Kähler manifold (on the results of Verbitsky). Geom. Funct. Anal..

[4] Green, M., Yoon-Joo, K., Laza, R., Robles, C.: The LLV decomposition of hyper-Kähler cohomology. arXiv:1906.03432 (2020)

[5] Huybrechts, D.: Compact hyperkähler manifolds. In: Calabi–Yau Manifolds and Related Geometries (Nordfjordeid. 2001), Universitext, pp. 161–225. Springer, Berlin (2003)

[6] Jacobson N (1951). Completely reducible Lie algebras of linear transformations. Proc. Am. Math. Soc..

[7] Looijenga E, Lunts VA (1997). A Lie algebra attached to a projective variety. Invent. Math..

[8] Markman E (2008). On the monodromy of moduli spaces of sheaves on $$K3$$ surfaces. J. Algebr. Geom..

[9] Oberdieck, G., Song, J.: Representation theory and cohomology (2021)

[10] Taelman, L.: Derived equivalences of hyperkähler varieties. arXiv:1906.08081 (2019)

[11] Verbitsky, M.: Action of the Lie algebra of $${\rm SO}(5)$$ on the cohomology of a hyper-Kähler manifold. Funktsional. Anal. i Prilozhen. Translation in Funct. Anal. Appl. **24**(3):70–71 (1990)

[12] Verbitsky, M.: Cohomology of compact hyperkaehler manifolds. PhD thesis at Harvard University (1995)

[13] Verbitsky, M.: Mirror symmetry for hyper-Kähler manifolds. In: Mirror Symmetry, III (Montreal, PQ, 1995), AMS/IP Stud. Adv. Math., vol. 10, pp. 115–156. Amer. Math. Soc., Providence (1999)

[14] Voisin, C.: Hodge theory and complex algebraic geometry. I, Cambridge Studies in Advanced Mathematics, vol. 76. Cambridge University Press, Cambridge, English edition (2007)

